# Alternative filter materials to natural sand and gravel in soil-based wastewater treatment systems

**DOI:** 10.1007/s11356-026-37831-8

**Published:** 2026-05-28

**Authors:** Erik Sindhøj, Elin Ulinder, Geert Cornelis, Andreas Lindhe, Ida Sylwan, Anna-Karin Dahlberg, Paul Löffler, David Eveborn, Jon-Petter Gustafsson, Karin Wiberg

**Affiliations:** 1 Department of Agriculture and Environmental Engineering, RISE – Research Institutes of Sweden, Uppsala, 75007 Sweden; 2 Department of Soil and Environment, Swedish University of Agricultural Sciences, Uppsala, 75007 Sweden; 3 Department of Architecture and Civil Engineering, Chalmers University of Technology, Gothenburg, 41296 Sweden; 4 IVL Swedish Environmental Research Institute, P.O. Box 210 60, Stockholm, 10031 Sweden; 5 Department of Aquatic Sciences and Assessment, Swedish University of Agricultural Sciences, Uppsala, 75007 Sweden; 6 Geological Survey of Sweden, Uppsala, 75236 Sweden

**Keywords:** On-site wastewater treatment, Soil treatment systems, Filter materials, Multi-criteria analysis, Sustainability assessment

## Abstract

Natural sand and gravel are widely used as filter media in soil treatment systems for decentralized wastewater treatment, but their extraction contributes to resource conflicts and environmental pressure. This study evaluated seven alternative filter materials: crushed rock, crushed concrete, washed excavated material, glacial till, bark, shredded tires, and biochar. A multi-criteria analysis was applied to assess technical performance, environmental sustainability, economic feasibility, and social acceptability. Bark showed strong environmental and social performance, while shredded tires and glacial till scored well in technical and economic criteria. Crushed concrete and washed excavated material performed similarly to natural gravel, whereas biochar scored lower due to high cost and uncertainty regarding long-term hydraulic and structural behavior. Across materials, trade-offs and data gaps were evident, particularly regarding long-term pollutant removal, leaching risks, and consistency of material quality. No single alternative outperformed natural gravel under all conditions. However, several materials demonstrated clear potential, provided that quality control measures are applied and field-scale validation is conducted. The applied multi-criteria framework provides a transparent basis for selecting sustainable filter materials and supports efforts to reduce dependence on natural gravel in decentralized wastewater treatment systems under context-specific conditions.

**Supplementary Information:**

The online version contains supplementary material available at 10.1007/s11356-026-37831-8.

## Introduction

Soil treatment systems (STS) are widely applied as decentralized wastewater treatment solutions in many parts of the world. In these systems, household wastewater is treated by percolation through soil or granular filter media, serving single households as well as small- to medium-sized communities. These systems are well established as robust treatment approaches, characterized by resilience to variable hydraulic and organic loading, limited operational and maintenance requirements, and comparatively low operating costs relative to mechanically intensive treatment technologies (Crites et al. 2014; Jenssen et al. 2005; Massoud et al. 2009). STS typically rely on natural sand and gravel as the primary filter medium, either for discharge to surface water or, in combination with additional filtration layers, for infiltration to groundwater.

Natural sand and gravel (hereafter referred to as natural gravel) are finite resources of strategic importance at the global scale (Bendixen et al. 2019; Peduzzi 2014). High-quality deposits often coincide with important groundwater systems used for drinking water supply (Torres et al. 2017). Growing concerns over resource sustainability, environmental impacts from extraction, and increasing pressure on protected groundwater reserves have intensified interest in identifying alternative filter media for STS. In addition, conventional STS filter materials may in some cases exhibit limited removal efficiency or be susceptible to clogging, further motivating the search for improved alternative substrates (Yang et al. 2018).

Suitable replacement materials must achieve comparable or superior treatment performance while removing key pollutants such as nutrients (e.g., phosphorus and nitrogen), pathogens, biochemical oxygen demand (BOD), pharmaceuticals, and microplastics. They must also meet requirements related to sustainability, cost-effectiveness, and material availability (Santos et al. 2024). Because STS operate under gravity-driven flow conditions, hydraulic conductivity and grain-size distribution are also critical design parameters, influencing loading capacity, clogging risk, and long-term system stability (Crites et al. 2014).

Natural gravel filters are widely reported to achieve high and stable removal of BOD and pathogens in soil-based wastewater treatment systems across a range of climatic and operational conditions (Crites et al. 2014; Wang et al. 2021). Phosphorus removal is more variable and strongly dependent on filter material properties, loading history, and sorption capacity, often declining over time as reactive sites become saturated (Bunce et al. 2018; Eveborn 2013; Hamisi et al. 2022). Nitrification can be effective in well-aerated sand and gravel filters, whereas total nitrogen removal is typically limited in single-pass systems without dedicated denitrification zones (Martikainen et al. 2018; Ritter & Eastburn 1988). Several studies have further shown that decentralized soil-based systems may achieve comparable or superior removal of certain organic micropollutants and pharmaceuticals relative to centralized wastewater treatment plants, owing to longer retention times and enhanced sorption and biodegradation processes (Ejhed et al. 2012; Heufelder 2010). Sand filtration has also been shown to retain a substantial fraction of microplastic particles (Sembiring et al. 2021; Wolff et al. 2021), although retention efficiency decreases with decreasing particle size (Pelley & Tufenkji 2008).

While the fundamental treatment processes in STS are broadly similar across regions, system design, performance expectations, and material suitability are strongly influenced by local regulatory frameworks, climatic conditions, and resource availability. National guidelines define effluent quality targets, design loading rates, and acceptable construction materials, thereby shaping both the baseline performance of conventional systems and the criteria against which alternative filter media are evaluated. Consequently, the relevance and feasibility of alternative materials must be assessed within specific national and regional contexts.

Crushed rock has emerged as a potential substitute for natural sand and gravel in several applications, including STS (Ulinder et al. 2021). In Sweden, where bedrock is geologically abundant, the suitability of crushed rock depends on achieving appropriate particle-size distributions and hydraulic properties. However, its widespread use may be constrained by production costs and environmental considerations. Internationally, a wide range of alternative filter materials has been investigated, including limestone, zeolites, dolomite, marine sediments, lightweight aggregates, and various plastics (Dacewicz & Chmielowski 2018; Johansson Westholm 2006). Growing interest in circular resource use has further stimulated the exploration of waste- and byproduct-based materials such as bark, wood chips, washed excavated material, slag, crushed concrete, and shredded tires.

This study aims to evaluate the potential of alternative filter materials to replace natural gravel in STS, using Sweden as a case study to assess material performance under well-defined regulatory conditions and high environmental protection standards. A multi-criteria analysis (MCA) framework was applied to assess candidate materials across technical, environmental, social, and economic sustainability dimensions. The assessment focuses on treatment performance, expected durability and clogging risk, and pollutant leaching risks, as well as broader considerations related to material availability, cost, and societal acceptance. The objective is to identify materials with strong potential for further development in Swedish applications rather than to designate a single optimal replacement.

The application of MCA in water and wastewater technology evaluation is well documented (e.g., Balkema et al. 2002; Neth et al. 2023). Sustainability-oriented MCA frameworks rely not only on measurable performance indicators but also on value judgments about which aspects of sustainability should be prioritized. Marques et al. (2015) emphasized that stakeholder perspectives are essential for defining what constitutes a sustainable water solution, since weighting choices inevitably reflect social, institutional, and contextual considerations. This perspective underpins the weighting approach adopted in this study, in which expert and stakeholder input was incorporated to balance multiple sustainability dimensions in a transparent and systematic manner. Compared with more common applications of MCA at the technology or system level, this study applies MCA to material substitution in decentralized sanitation infrastructure.

This study goes beyond a conventional literature review by applying a structured MCA framework to evaluate alternative materials. The scope of the study is limited to single-material replacements for natural gravel used in filter beds or reinforcement layers in STS. Reactive polishing materials (e.g., phosphorus traps) and alternative STS system components (e.g., modular drainage or distribution layers) are not included. Only materials considered to be locally available in Sweden at a scale relevant for practical implementation and not posing a significant environmental risk were included in the assessment.

## Material and methods

**Fig. 1:**
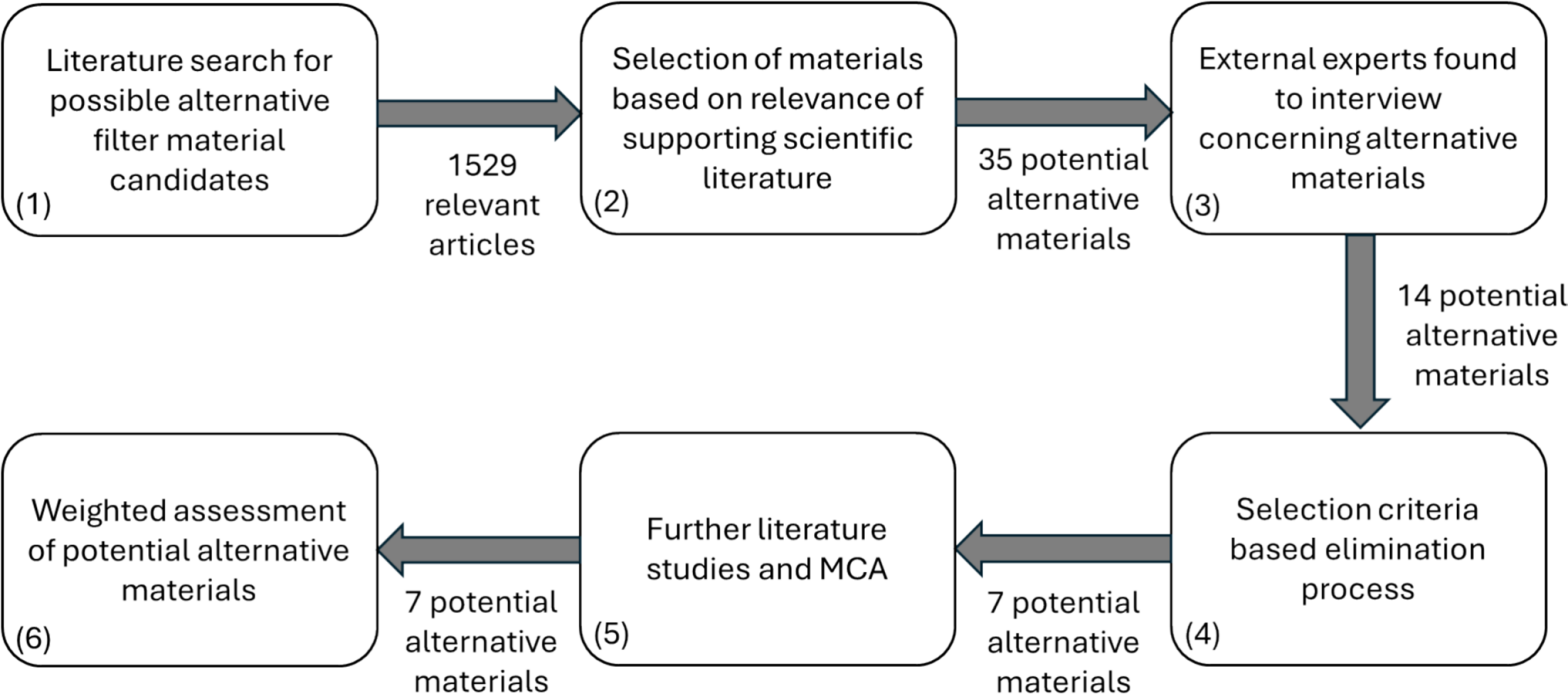
Stepwise workflow used to screen and select candidate alternative filter materials for replacement of natural sand and gravel

This study developed and applied a systematic and multi-phased methodology to identify, evaluate, and compare alternative filter materials to natural sand and gravel for use in STS. The process, illustrated in Fig. 1, included literature search and sorting (Step 1–3), expert consultation (Step 4), database development, performance analysis, and MCA evaluation (Step 5–6).

### Identification of candidate materials (Step 1–3)

An initial screening of potential filter materials was performed through an extensive literature search in Scopus. The search string was designed to be broad and inclusive, incorporating 76 terms related to filter media, filtration, infiltration, drainage, wastewater, sewage, septic, blackwater, stormwater, seepage bed, percolation, land treatment, and soil treatment systems.

In the second screening step (Fig. 1), articles were manually reviewed for evidence of effectiveness in removing organic matter and pathogens. Materials were then grouped into three categories: natural minerals, processed or recycled materials, and bio-based materials. A third screening step retained only materials for which an expert or industry contact familiar with the material could be identified. This criterion was applied to facilitate access to unpublished expert knowledge, grey literature (e.g., reports and facts sheets), and insights into national availability and practical use.

### Expert consultation and extended review (Step 4)

A structured expert consultation process was carried out to support material screening, data interpretation, and subsequent development of the MCA. Expert input was gathered through a series of workshops and targeted interviews involving participants from research institutes, public authorities, and industry. These included researchers and practitioners with expertise in soil-based wastewater treatment systems, environmental engineering, filter material performance, recycled and mineral construction materials, and environmental risk assessment.

The expert consultations served several purposes. First, it helped identify relevant grey literature and supplementary data sources not captured through the scientific database search. Second, it provided contextual information on material performance, known technical risks, expected challenges for use as filter media, geographic availability, transportability, production volumes, and practical experience with material handling and quality variation. Third, it supported interpretation of data gaps and uncertainties in cases where published evidence was limited or heterogeneous.

The workshops and seminars were structured around presentations of preliminary findings and candidate materials, followed by moderated discussions in which experts were invited to comment on material suitability, potential limitations, and practical feasibility under Swedish conditions. Targeted interviews were conducted when more material-specific knowledge was needed. The aim of this consultation was not to establish a formal consensus, but to strengthen the robustness of the screening process and ensure that the subsequent MCA reflected both published evidence and current professional knowledge.

This expert consultation was complemented by a deeper literature review focusing on pollutant removal efficiency, potential leaching of inorganic and organic contaminants, climate impact, and other key performance characteristics. In addition, the potential for material decomposition and suitability as a standalone replacement for natural gravel were considered. Based on these combined inputs, the list of materials was reduced to a shortlist of seven candidate materials for in-depth evaluation.

As noted above, the study focused exclusively on materials that could function as the primary filtration layer in soil treatment systems. Reactive materials designed primarily for targeted pollutant removal, such as phosphorus sorbents, were therefore excluded at this stage. The purpose of this step was to identify materials with sufficient technical relevance and sustainability potential for further comparative assessment.

### Multi-criteria analysis (MCA) (Step 5)

The MCA was applied as an integrative decision-support framework to systematically compare alternative filter materials across multiple sustainability dimensions. The MCA translates heterogeneous quantitative and qualitative information into a structured assessment by defining explicit criteria, indicators, and scoring rules. It allows for the inclusion of uncertainty and expert judgement where empirical data are limited. In this study, the MCA synthesized evidence on technical performance, environmental sustainability, economic feasibility, and social acceptability into a transparent and comparable evaluation of candidate materials relative to natural gravel.

#### MCA framework, scoring, and uncertainty

**Fig. 2:**
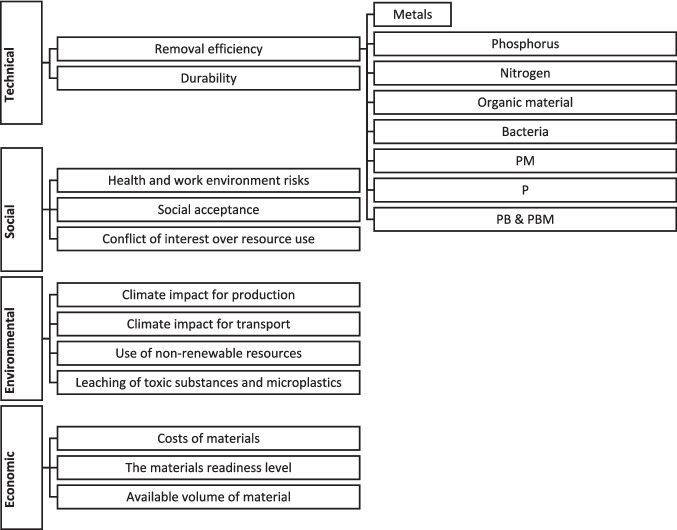
Structure of the multi-criteria analysis (MCA) framework, illustrating the four main criteria and their associated sub-criteria, including pollutant groupings used to assess technical performance

The MCA framework evaluated each material against four main criteria: (1) technical performance, (2) environmental sustainability, (3) economic feasibility, and (4) social acceptability. Within the technical criterion, particular emphasis was placed on pollutant removal performance, including bacteria and viruses as indicators of pathogen-related treatment performance. Additional emphasis was placed on expected lifespan and clogging risk as indicators of long-term durability. Each criterion was operationalized through a set of sub-criteria and indicators, as summarized in Fig. 2.

Materials were scored relative to natural gravel using a seven-point scale ranging from −3 (major negative effect) to +3 (major positive effect), with 0 indicating performance equivalent to natural gravel. Scores of −2 and +2 represent moderately negative or positive effects, while scores −1 and +1 represent slightly negative or positive effects. Uncertainty associated with each score was classified into four levels based on the consistency, quality, and availability of supporting data: low (  ), moderate (  ), high (  ), to very high (  ).

The assessments were based primarily on the in-depth literature review and were supplemented, where relevant, by expert judgement generated through the consultation process described in Step 4. Expert input was used particularly to support interpretation of qualitative criteria and to assess materials in areas where published evidence was sparse, highly variable, or context dependent. At this stage, the MCA focused on characterizing material performance under each criterion. The relative importance of criteria was addressed subsequently through a dedicated weighting procedure.

#### Technical performance

##### Data collection and assessment with respect to technical performance

A targeted search for each shortlisted material was conducted using Scopus, Google Scholar, and supplementary references identified through the expert consultation process. Data were compiled from 60 experimental studies, including column tests, pilot-scale setups, and full-scale systems. Batch studies were excluded due to their limited relevance to real-world filtration conditions.

Collected parameters included hydraulic conductivity, pollutant removal efficiency, particle characteristics, and test conditions. The particle-size metrics d_10_, d_50_, and d_60_ represent the grain diameters at which 10%, 50%, and 60% of the sample mass pass through the sieve, respectively. These metrics are commonly used to describe grain-size distribution and calculate the uniformity coefficient. Table 1 summarizes the metadata fields extracted from each study and included in the compiled database.

**Table 1:** Parameters and metadata collected from 60 experimental studies included in the technical performance assessment

Parameter	Unit
Mass of filter material	kg
Porosity	-
d_10_	mm
d_50_	mm
d_60_	mm
Bulk density	g cm^−3^
Test scale	Column, pilot, full-scale
Infiltration length (column test)	m
Flow area	m^2^
Flow rate	L h^−1^
Test duration	Days
Hydraulic conductivity	m s^−1^
Influent water	Stormwater, domestic or artificial WW
pH	-
Input concentration (of different substances)	mg L^−1^
Output concentration (of different substances)	mg L^−1^
Reported removal efficiency (of different substances)	%

For removal efficiency and hydraulic conductivity, scoring and uncertainty assessments were based on quantitative data compiled from the literature. Where long-term performance or material suitability could not be fully assessed from published studies alone, expert input (described in Step 4) was used to support qualitative interpretation, particularly for lifespan, clogging risk, and practical feasibility.

##### Hydraulic conductivity

Hydraulic conductivity (*Kₛ*) data were compiled from 24 published studies, yielding 66 directly measured values. An additional 10 values were estimated from particle-size distribution data using Breyer’s empirical equation (Eq. 1). The full list of source studies is provided in the Supplementary Information (Table S1). Reported values ranged across several orders of magnitude (4.8 × 10⁻⁸ to 7.4 cm s⁻^1^). Therefore, all data were log-transformed (log₁₀ *Kₛ*) prior to comparison to enable clearer interpretation across materials.

When not reported, hydraulic conductivity (*K*_*s*_) was estimated from grain size distribution using Breyer’s empirical equation:

1 $${K}_{s}={C}_{B}\frac{g}{\nu }log\left(\frac{500}{U}\right){d}_{10}^{2}$$

where *C*_*B*_ is Breyer’s constant (6 × 10^–4^), *g* = gravitational acceleration (9.81 m s^−2^), *v* is the kinematic viscosity at 25 °C (0.89 × 10–6 m^2^ s^−1^) and *U* is the uniformity coefficient (d_60_/d_10_). The parameters d_10_ and d_60_ represent the particle diameters at which 10% and 60% of the material mass pass through the sieve, respectively. This method has been shown to provide strong agreement with cylinder tests measurements (Wang et al. 2017).

##### Pollutant removal efficiency

Treatment capacity is typically evaluated through column tests or similar experiments, where measured influent and effluent concentrations are used to calculate removal efficiency and assess how a material is likely to perform in full-scale systems. The most reported parameter is removal efficiency (*R*), calculated as:

2 $${R}_{m}=\frac{{C}_{in}-{C}_{out}}{{C}_{in}}$$

where *C*_*in*_ is the input concentration and *C*_*out*_ is the outlet concentration integrated over a certain experimental time. *R* for a particular material (*R*_*m*_) is straightforward to calculate but varies over time due to surface saturation and biofilm formation on the filter material. Additionally, *R*_*m*_ depends on experimental setup factors such as column dimensions, flow rate, and inlet concentration, making it difficult to directly extrapolate to full-scale applications.

To ensure comparability across materials and studies, pollutants were grouped based on shared transport and persistence characteristics. Removal efficiency data were collected for the pollutant groups shown in Table 2, based on published studies reporting paired influent and effluent concentrations. The complete dataset used in the analysis is provided in the Supplementary Information (Table S2).

**Table 2:** Pollutant groups for which removal efficiency data were collected and their classification used in the MCA. Organic micropollutants were further classified according to persistence (P), bioaccumulation potential (B), and mobility (M)

Pollutant group	Pollutants
Metals	Cu, Zn, Pb, Ni, Cd, Cr, As
Phosphorus	Total P, orthophosphate, organic phosphorus
Nitrogen	Ammonium, nitrate, nitrite, total N
Organic material	COD, BOD, TOC
Bacteria and viruses	E. coli, faecal coliforms, total coliforms, enterococci, viral counts
Organic micropollutants	
PB and PBM	PBnM, PBM
PM	PMnB
P	PnMnB

Pollutants were grouped by similar behavior during filtration to reduce complexity of the MCA. This grouping also considered the limited availability of data, as considering individual pollutants would in many cases result in sparse datasets. Organic micropollutants were further classified by persistence (P), mobility (M), and bioaccumulation potential (B), following Neumann and Schliebner (2019) and European Commission (2006). PBM for respective pollutants were predicted using EPI Suite software v4.11 (US EPA, Washington, DC), with pollutants categorized into three levels: non- (prefix “n-”), normal (no prefix), and strong (prefix “v-”). Pollutants were then grouped as follows:

P: persistent, non-mobile, non-bioaccumulative

PM: persistent and mobile, non-bioaccumulative

PB and PBM: persistent and bioaccumulative and mobile

Persistence was estimated based on degradation half-life, mobility using the organic carbon-water partition coefficient, and biodegradation via the bioconcentration factor. Non-persistent pollutants were excluded as they degrade quickly in nature and thus pose limited long-term environmental risk. Microplastics were also excluded due to a lack of removal efficiency data for most considered filter materials.

The accumulated mass of removed pollutants per unit material (*S*_*m*_, mg g^−1^) was estimated for the duration of the experiment based on reported influent concentrations, flow rates, operational time, average removal efficiencies, and mass of filter material used. This provided an approximate indication of each material’s long-term adsorption capacity under the conditions studied, calculated as:

3 $${S}_{m}=\frac{{C}_{in}\times {R}_{m}\times Q\times t}{m}$$

where *C*_*in*_ is the average influent concentration [mg L^−1^], *Q* is the flow rate [L day^−1^], *t* is the operational duration [days], *R*_*m*_ is the removal efficiency (Eq. 4), and *m* is the mass of filter material used during the experiment. The resulting value represents the cumulative mass of pollutants removed per mass of filter material during the study period.

Although adsorption capacity (${S}_{m}$) provides an indication of potential long-term pollutant retention, removal efficiency was selected as the primary input to the MCA because it is the most consistently reported parameter across studies. It also reflects the combined effect of sorption, filtration, biological degradation, and other removal mechanisms relevant to STSs. In most published experiments, *Sm* cannot be reliably compared across materials because test durations, influent concentrations, and column configurations vary widely. These factors strongly influence the accumulated mass removed per unit material. Removal efficiency, although also influenced by experimental design, provides a more comparable measure of short-term functional performance. It therefore serves as a practical proxy for how a material is likely to behave under field conditions.

Adsorption capacity was still calculated and used as supporting information to interpret long-term trends but it was not incorporated directly into the MCA due to inconsistent reporting, limited data availability for several materials, and the difficulty of harmonizing test conditions across the literature. The calculated *S*_*m*_ values used to support interpretation of long-term retention potential are provided in the Supplementary Information (Table S2).

##### Treatment performance relative to natural gravel

Natural gravel was used as the reference material for technical performance, reflecting its widespread use as the baseline filter medium in STS. Treatment performance of alternative filter materials was therefore evaluated relative to natural gravel using a relative removal efficiency index (*RRE*), calculated separately for each pollutant group (*p*).

Removal efficiencies for a given material (*R*_*m*_) were quantitatively compared with those of natural gravel (*R*_*ng*_) and translated into a seven-category scoring scale ranging from −3 to +3, where 0 represents performance equivalent to natural gravel. The RRE was calculated using the following equations:

4 $${RRE}_{p}=round\left(\frac{{R}_{m}-{R}_{ng}}{{R}_{ng} \cdot \frac{1}{3}}\right) if {R}_{m}< {R}_{ng}$$

5 $${RRE}_{p}=round\left(\frac{{R}_{m}-{R}_{ng}}{(1-{R}_{ng}) \cdot \frac{1}{3}}\right) if {R}_{m}> {R}_{ng}$$

Here, *R*_*m*_ and *R*_*ng*_ represent the removal efficiencies of the alternative material and natural gravel, respectively. The factor of 1/3 defines category widths corresponding to differences of approximately 33 percentage points in relative performance. For example, an absolute difference between *R*_*m*_ and *R*_*ng*_ of less than 33% was assigned a score of 0, while a difference of −40% was assigned a score of −1 when *R*_*ng*_ > *R*_*m*_. When no removal efficiency data were available for a given material and pollutant group, *RRE*_*p*_ was conservatively set to 0, indicating performance equivalent to natural gravel.

#### Environmental sustainability

Environmental sustainability was assessed using indicators related to climate impact, resource use, and potential environmental risks associated with material production and use. The assessment focused on cradle-to-gate impacts to ensure comparability across materials and to avoid introducing uncertainty related to installation, operation, or end-of-life stages, which are highly site-specific for soil treatment systems.

Climate impact was primarily assessed using Global Warming Potential (GWP) values derived from published life cycle assessment (LCA) studies for the respective materials. When peer-reviewed or publicly available LCA data were unavailable or insufficient, Environmental Product Declarations (EPDs) were used as a secondary data source to provide indicative estimates. Only functionally comparable data were included, and results were interpreted comparatively rather than as absolute impact values.

Potential environmental risks were evaluated qualitatively, with particular emphasis on the likelihood of leaching harmful inorganic or organic substances during long-term contact with wastewater. This included heavy metals, organic additives, and other material-specific contaminants reported in the literature. Where empirical leaching data were limited, inconsistent, or context-dependent, uncertainty scores were increased accordingly to reflect data gaps and avoid overconfidence in the assessment.

#### Economic feasibility

Economic feasibility was assessed based on the relative costs associated with producing, processing, and supplying each filter material. The assessment focused on material costs at the point of delivery, including extraction or recovery, processing, and transport where relevant. Installation and maintenance costs were not explicitly included, as these are largely system specific and expected to be similar across granular filter materials used in comparable STS designs.

Cost data were compiled from multiple sources, including supplier price lists, quarry and recycling facility price information, and published market reports. Due to substantial regional variability and differences in production scale, cost estimates were treated as indicative rather than absolute values and were used for relative comparison within the MCA. Uncertainty in the economic assessment was explicitly considered, with higher uncertainty assigned to materials for which price data were sparse, outdated, or highly context dependent, such as byproducts and waste-derived materials with limited or emerging markets.

#### Social acceptability

Social acceptability was evaluated qualitatively using indicators related to occupational health and safety, resource-use conflicts, and societal acceptance of alternative filter materials. The assessment considered potential risks during material handling, processing, and installation, as well as broader societal perceptions related to the use of waste-derived or industrial byproduct materials in wastewater treatment applications.

Because standardized quantitative indicators for social performance in decentralized wastewater infrastructure are limited, this dimension relied on a combination of literature evidence, expert judgement, and stakeholder input obtained through the consultation process described in Step 4. Expert input was used to contextualize likely acceptance barriers, identify practical health and work-environment issues, and support scoring where empirical evidence was limited. Materials associated with potential health risks, conflicting land or resource use, or low public acceptance were assigned lower scores and higher uncertainty.

Uncertainty scores were used to explicitly reflect the subjective nature of social assessments and the variability in societal acceptance across regions and stakeholder groups. Where empirical evidence or documented experience was lacking, uncertainty was increased to avoid overconfidence in the assigned scores.

### Criteria weighting and aggregation (Step 6)

Weighting of criteria is a critical step in MCA, as it reflects value judgements regarding the relative importance of different sustainability aspects and can strongly influence overall rankings. Recent work has highlighted the need for transparency and robustness in weighting procedures, particularly in sustainability assessments that combine technical, environmental, economic, and social dimensions (Neth et al. 2023). In line with these recommendations, weighting in this study was applied after scoring and was informed by expert and stakeholder input. A sensitivity analysis was conducted to evaluate how alternative weighting assumptions affect the results.

Sub-criteria importance was elicited using direct rating on a 0–10 scale, where higher values indicate greater perceived importance. Initial ratings were obtained from the project expert team, which included researchers and practitioners involved in the screening and assessment process described in Step 4. These ratings were then presented and discussed during a validation workshop with a broader stakeholder reference group.

The stakeholder reference group included representatives from national water authorities, wastewater treatment sector, the aggregates and construction materials industry, and the research community. The purpose of the workshop was to review the coherence of the weighting structure, identify any evident imbalances, and refine the ratings where clarification was needed. This process did not aim to generate formal consensus weights, but rather to improve the transparency, credibility, and contextual relevance of the MCA.

To derive weights suitable for aggregation, the elicited importance ratings were normalized. For each main criterion *h*, sub-criterion weights were calculated by normalizing the sub-criterion rating *s*_*h,k*_ by the sum of ratings within that main criterion:

6 $${w}_{h,k}=\frac{{s}_{h,k}}{\sum_{k=1}^{{K}_{h}}{s}_{h,k}}$$

where *w*_*h,k*_ is the normalized weight of sub-criterion *k* under main criterion *h*, and *K*_*h*_ is the number of sub-criteria within criterion *h*. This yielded normalized sub-criterion weights that sum to 1 within each criterion.

Main criterion weights were derived analogously by normalizing the importance rating s_*h*_ assigned to the four main criteria:

7 $${W}_{h}=\frac{{s}_{h}}{\sum_{k=1}^{H}{s}_{h}}$$

where *W*_*h*_ is the normalized weight of main criterion *h*, and *H* is the number of main criteria. This yields normalized criterion weights that sum to 1 across the full MCA.

A hierarchical weighting procedure was applied by propagating the main-criterion weights to the sub-criterion level. The global weight of each sub-criterion was therefore:

8 $${v}_{h,k}={W}_{h}\cdot {w}_{h,k}$$

This ensures that both (i) the relative importance of sub-criteria within a main criterion and (ii) the relative importance of the main criteria in the overall assessment are consistently reflected in the aggregation.

#### Calculation of weighted scores

A linear additive model was used to calculate weighted scores according to the following procedure. Performance scores for each material *a* were assigned for each sub-criterion *k* under main criterion *h* using the MCA scoring scale (−3 to +3) relative to natural gravel (0). Let *p*_*a,h,k*_ denote the score for material *a* on sub-criterion *k* within main criterion *h*.

Sub-criterion scores were first aggregated to criterion-level scores using a linear additive model:

9 $${P}_{a,h}=\sum_{k=1}^{{K}_{h}}{w}_{h,k}\cdot{p}_{a,h, k}$$

The overall weighed MCA score for each material was then calculated as:

10 $${P}_{a}=\sum_{h=1}^{H}{W}_{h}{P}_{a,h}$$

where *P*_*a*_ is the overall score for material *a*, and *P*_*a,h*_ is the aggregated score for main criterion *h*.

A sensitivity analysis was conducted to examine how alternative plausible weighting assumptions influenced the overall ranking of materials.

## Results and discussion

The literature search (Step 1) resulted in 1529 articles. After an initial screening of abstracts, around 900 were excluded as non-relevant. Of the remaining articles, approximately 350 were assessed in more detail to determine whether they provided evidence of treatment performance relevant for STS applications (Step 2). This process resulted in a list of 35 candidate filter materials with potential to replace natural gravel (Table 3). These were grouped into three categories: natural minerals, processed or recycled, and bio-based materials.

**Table 3:** Filter materials identified through the literature review (Step 1) and retained after initial screening (Step 2), grouped by material origin into natural mineral, processed or recycled, and bio-based materials (*n* = 35)

Natural mineral	Processed or recycled materials	Bio-based materials
Crushed rock	Iron ore tailings sand	Bark
Dolomite	Washed excavation material	Biochar
Limestone	Plastic biomodules	Sheep wool
Sea sand	Expanded polystyrene chips	Coconut shell
Shale	Shredded tires	Mussel shells
Glacial till	Glass wool	Peat
Perlite	Calcined limestone	Wood fleece
Vermiculite	Crushed concrete	Cloth materials
Wollastonite	Crushed terracotta	
Zeolites	Crushed glass	
	Crushed bricks	
	Light expanded clay aggregates (LECA)	
	Polonite	
	Slag	
	Mineral wool	
	Grit chamber sand from WWTPs	
	Filtraflo-p	

The next sorting criterion for materials was the availability of industry or academic experts who could provide additional contextual information and help identify relevant gray literature (Step 3, see Fig. 1). Experts were found for 14 of the 35 materials (Table 4). These 14 materials were subjected to further evaluation through workshops and interviews, supported by deeper literature reviews.

**Table 4:** Materials retained for in-depth evaluation (Step 3) based on the availability of treatment performance data and identification of relevant experts for consultation. Numbers in parentheses indicate the number of articles identified for each material in the initial literature search

Natural mineral	Processed or recycled materials	Bio-based materials
Crushed rock (6)	Washed excavation material (4)	Bark (10)
Glacial till (2)	Plastic biomodules (26)	Biochar (83)
Zeolites (86)	Shredded tires (7)	Sheep wool (0)
	Crushed concrete (13)	Coconut shell (16)
	Slag (37)	Mussel shells (21)
		Peat (32)

During Step 4, several materials were excluded due to practical, regulatory or sustainability constraints. Zeolites, despite well-documented adsorption properties, were excluded because no domestic production exists in Sweden, making their use dependent on long-distance transport. Plastic biomodules were excluded due to disposal challenges and concerns about microplastics release in the environment. Slag has demonstrated strong phosphorus-binding capacity but generally requires combination with other media to support biological treatment and BOD removal (Johansson Westholm 2006). Sheep wool was excluded due to risk for nitrogen release during decomposition (Camilli et al. 2025). Coconut shells were excluded, despite evidence of effectiveness as an STS filter material (Brown et al. 2025; Kumar et al. 2025), because they are not locally available. Mussel shells were excluded due to uncertainties with the Animal Byproduct Regulation in Sweden and limited availability.

Following this evaluation, seven materials were shortlisted for in-depth evaluation (Table 5). These represent the materials with sufficient data availability, stakeholder knowledge, and potential to function as standalone filter media in the primary filtration layer of STSs.

**Table 5:** Final shortlist of filter materials retained for in-depth analysis and evaluation in the multi-criteria assessment (Step 4)

Natural mineral	Processed or recycled materials	Bio-based materials
Crushed rock	Washed excavation material	Bark
Glacial till	Shredded tires	Biochar
	Crushed concrete	

Bulk density values for the short-listed materials were compiled as these influence several technical, environmental, and economic criteria used in the subsequent MCA (Table 6).

**Table 6:** Bulk density ranges for natural gravel and shortlisted alternative filter materials used in the technical and economic assessments

Material	Bulk density (t m^−3^)	Reference
Natural gravel	1.5–1.6	AB Nybrogrus^a^; Snabb Grus^b^; Heidelberg Materials^c^
Crushed rock	1.5–1.6	AB Nybrogrus^a^; Snabb Grus^b^; Swerock^d^
Glacial till	1.6–2.1	SGI 1970; Yin et al. 2018
Washed excavation material	1.5–1.6	Assumed similar to natural gravel and crushed rock
Shredded tires	0.35–0.36	Andreas Pettersson, Personal Communication, 2022-08−25
Crushed concrete	1.5–1.65	AB Nybrogrus^a^; Hummeltorp^e^
Bark	0.25–0.45	Heidelberg Materials^c^; Zugol^f^
Biochar	0.08–1.1	Brewer and Levine 2015; Alhashimi and Aktas 2017

^a^Data obtained from publicly available supplier information from AB Nybrogrus, accessed 15 May, 2024

^b^Data obtained from publicly available supplier information from Snabb Grus, accessed 15 May, 2024

^c^Data obtained from publicly available supplier information from Heidelberg Materials, accessed 15 May, 2024

^d^Data obtained from publicly available supplier information from Swerock, accessed 15 May, 2024

^e^Data obtained from publicly available supplier information from Hummeltorp, accessed 15 May, 2024

^f^Data obtained from publicly available supplier information from Zugol, accessed 15 May, 2024

### Technical criteria

#### Hydraulic conductivity

**Fig. 3:**
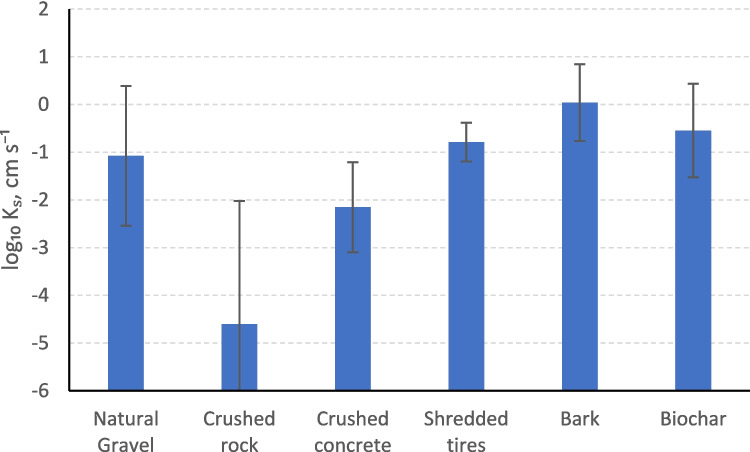
Log 10 transformation of hydraulic conductivity (*Kₛ*, cm s⁻^1^) of the filter materials based on published measurements and estimates derived from grain-size data. Bars show mean values and error bars represent ±1 standard deviation across studies

Because hydraulic behavior influences long-term stability and clogging risk, hydraulic conductivity was evaluated separately to provide contextual information on initial permeability among the shortlisted materials. Reported hydraulic conductivity values varied by several orders of magnitude, both within and between materials, reflecting differences in grain size distribution, test configuration, and material preparation (Fig. 3).

When expressed on a log_10_ scale, most materials showed hydraulic conductivity ranges that overlapped with those reported for natural gravel-based filters in Scandinavia field installations (Ulinder et al. 2021). Crushed rock and crushed concrete tended to show lower average hydraulic conductivity, particularly where particle size distributions were more heterogeneous, which can reduce pore connectivity and restrict flow (Nguyen et al. 2022; Ulinder et al. 2023). However, substantial variability was observed, and overlapping values with natural gravel were present for all materials (Fig. 3).

Bark and biochar showed relatively high initial hydraulic conductivity on average but also considerable variability. For these materials, changes in pore structure due to degradation, compaction, or fragmentation may alter hydraulic behavior over time (Krona 2017; Markiewicz et al. 2020). Industry observations further suggest that bark filters may require replenishment, approximately 15% of the filter volume, every 10 years due to decomposition, although this could depend on wastewater composition (S. Maunoir, personal communication, September 12, 2022). In addition, discoloration and elevated biochemical oxygen demand (BOD) have been noted in effluents from bark-based filters, indicating leaching of organic substances (Ribé et al. 2009). Biochar also displayed relatively high initial permeability, although its potential to compact or fragment could alter its hydraulic behavior during extended use. For both materials, the limited availability of long-term permeability data contributes to moderate uncertainty. Overall, the wide range of reported values suggests that hydraulic conductivity is strongly influenced by material preparation and system design rather than material type alone.

Hydraulic conductivity was therefore treated as background information and not included as a scored criterion in the MCA, as both higher and lower permeability can be advantageous or disadvantageous depending on system configuration, loading conditions, and treatment objectives.

#### Durability and clogging risk

Durability was interpreted as the expected lifespan of each material under unsaturated wastewater flow, considering structural stability, susceptibility to clogging, and known degradation processes. Because long-term operational data are limited for most alternatives, the assessments rely on short-term experimental findings, material characteristics, and practical experience from related filtration applications. A quantitative durability constant could not be derived because comparable long-term degradation data were not available across materials. Durability was therefore assessed comparatively using reported material behaviour, structural stability, and practical evidence.

Similar relationships between substrate structure, clogging risk, and long-term stability have been documented in constructed wetland media, where small particle sizes, low porosity, and poor hydraulic conductivity are consistently identified as major drivers of clogging and declining performance over time (Yang et al. 2018). These findings support the relevance of particle-size characteristics as indicators of durability under unsaturated flow conditions.

**Fig. 4:**
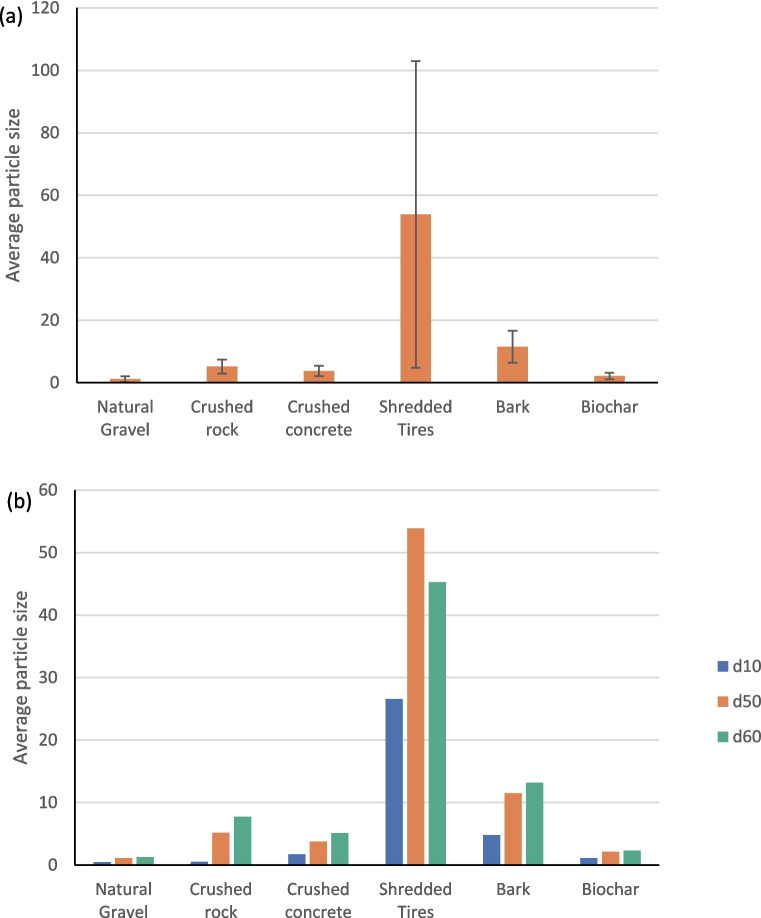
Particle-size characteristics of the filter materials. **a** Mean particle size expressed as d₅₀, with error bars representing ±1 standard deviation across studies.﻿ **b** Grain-size percentiles (d₁₀, d₅₀, d₆₀), illustrating differences in particle-size distribution and sorting between materials

Although the particle-size evaluation was based on a limited number of observations, some general insights can be drawn regarding the particle size distribution of the materials. The average particle size was similar for most materials, with the exception of bark and shredded tires, which showed larger mean grain sizes and a substantially wider spread, particularly for shredded tires (Fig. 4A). A closer examination of particle-size percentiles (d₁₀, d₅₀, d₆₀) reveals clearer differences in particle-size structure between materials (Fig. 4B). These values represent the grain diameters at which 10%, 50%, and 60% of the sample mass pass through the sieve, respectively. The uniformity (d_60_/d_10_) indicates how well sorted or heterogeneous a material is.

Crushed rock and crushed concrete showed relatively high uniformity coefficients, averaging 20 and 12, respectively, indicating heterogeneous grain-size distributions. This likely contributes to their relatively low hydraulic conductivity, as increased heterogeneity can reduce pore connectivity and effective porosity. In contrast, other materials showed average uniformity coefficients between 2.2 and 5.1, corresponding to more uniform grain-size distribution and more consistent hydraulic behavior. These patterns in particle-size distribution complement the qualitative durability assessments, as clogging risk and long-term hydraulic stability are strongly influenced by the abundance of fines, grain-size heterogeneity, and the potential for particle breakdown or biofilm accumulation.

Washed excavated material, crushed rock, shredded tires, crushed concrete, and glacial till were all assessed as having lifespans broadly comparable to natural gravel. Their mineralogical stability and relatively robust grain-size distributions suggest low susceptibility to physical breakdown. These materials also share similar resistance to compaction, implying that their hydraulic properties are likely to remain stable over time. However, the scarcity of long-term studies in onsite wastewater treatment means that the lifespan assessments carry moderate to high uncertainty.

Two materials showed clearer risks of degradation. Bark is prone to gradual decomposition, especially in contact with nutrient-rich wastewater, which can generate fine particles that reduce permeability and promote localized clogging. Experiences from bark-based filters in other treatment contexts indicate that material replenishment may be necessary over time (Krona 2017). Biochar, while chemically stable, is brittle and may compact under load, which can reduce pore space and limit flow. So, while short-term tests indicate that both materials can function effectively when freshly installed, they received lower durability estimates.

Durability and clogging risk are closely linked to hydraulic performance, as changes in pore structure from particle breakdown, compaction, or biofilm accumulation can influence long-term flow conditions. These interactions mean that durability should be interpreted together with hydraulic conductivity to understand how a material may perform throughout its service life.

#### Removal efficiency

In total, 730 experimental measurements of influent and effluent concentrations were identified across 60 studies involving the seven filter materials. All individual measurements and associated source references are provided in Supplementary Information (Table S2). From these, removal efficiencies (*Rm*) were calculated using Eq. 2. Mean removal efficiencies were determined for each material and pollutant group (Table 7). Each material’s relative removal efficiency (*RRE*_*m*_) compared to natural gravel was assessed according to Eqs. 4 and 5 and used for the MCA scoring along with the associated uncertainty (Table 8). Data coverage varied across materials and contaminant types.

**Table 7:** Mean removal efficiency (*Rₘ*, %) for different pollutant groups across filter materials, reported as mean ±1 standard deviation based on compiled literature data. Washed excavated material and glacial till were excluded due to insufficient removal efficiency data. “NA” indicates that no suitable data were available for the respective material-pollutant combination

Pollutant group	Crushed rock	Crushed concrete	Shredded tires	Bark	Biochar	Natural gravel
Organic material	64 (±23)	55 (±31)	64 (±36)	77 (±15)	68 (±25)	74 (±23)
Bacteria	87 (±23)	99 (NA)	91 (±38)	54 (±49)	81 (±38)	73 (±33)
Nitrogen	17 (±23)	38 (±37)	48 (±41)	20 (±30)	25 (±141)	20 (±30)
Phosphorus	28 (±23)	34 (±30)	69 (±36)	63 (±42)	27 (±52)	53 (±38)
Metals	NA	NA	NA	NA	2 (±1)	92 (±5)
PM	5 (±23)	NA	42 (±7)	76 (±35)	53 (±38)	33 (±36)
P	NA	NA	NA	91 (±20)	90 (±12)	99 (±2)
PB and PBM	NA	NA	NA	99 (±3)	91 (±10)	99 (±2)

*P, persistent; *B*, bioaccumulative; *M*, mobile

**Table 8:**
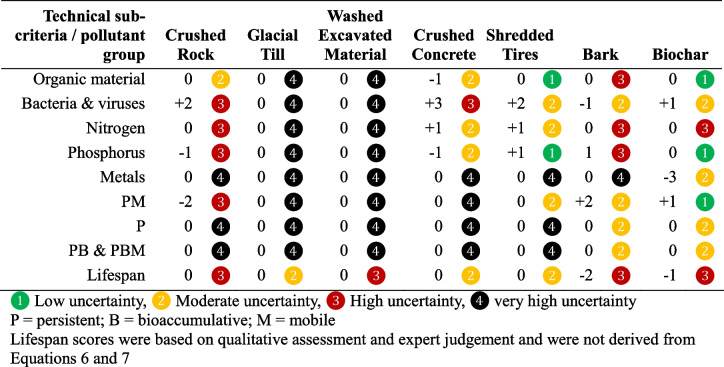
Relative technical performance scores (*RREₘ*) for each filter material compared to natural gravel (0), based on Eqs. 4 and 5, with associated uncertainty classes. Scores are reported for individual technical sub-criteria and pollutant groups

A general observation is that most materials showed removal efficiencies similar to natural gravel across several pollutant groups. However, considerable variability in experimental setup (e.g., column, pilot, or full-scale systems), design, influent characteristics, and test durations resulted in high uncertainty for many of the assessments. For washed excavated materials and glacial till, no specific data on removal efficiency was found. However, both materials were assessed as likely to perform similarly to natural gravel, based on their mineralogical composition and grain size distribution. However, this assessment carries very high uncertainty for washed excavated materials and lower uncertainty for glacial till due to its closer similarity to gravel.

For materials where data was available, bark, crushed rock, and biochar showed removal efficiencies similar to natural gravel for most pollutants. Data gaps were present, particularly for metals (bark and crushed rock) and organic micropollutants (crushed rock). Biochar demonstrated higher variability in performance, reflecting differences in production methods and feedstock. While biochar has shown strong adsorption capacity for metals in some laboratory studies, this effect is often limited to modified biochars, whereas unmodified biochar shows more variable results.

Shredded tires also lacked data for metals and many micropollutants. Although available studies indicate that their removal efficiency for organic matter and nutrients can be comparable to natural gravel, several reviews have noted risks of leaching zinc and rubber-derived organic compounds (Yang et al. 2018). Similarly, crushed concrete showed removal efficiency comparable to natural gravel based on the available data, although data were missing for several pollutant groups. Overall, the highest uncertainties were associated with materials for which specific filtration studies were lacking or where tested material properties varied substantially between studies.

#### Long-term removal capacity

Long-term removal capacity is an important aspect of filter material performance, as it reflects the cumulative ability of a material to retain pollutants over time under continuous loading. In filtration studies, this is commonly expressed as the accumulated mass of removed pollutants per unit material (*S*_*m*_), which integrates the effects of adsorption, filtration, and biological degradation processes.

To explore this aspect, *S*_*m*_ was calculated for materials where sufficient data were available, using Eq. 2. The resulting values (Table 9) should be interpreted as indicative rather than directly comparable, as they are strongly influenced by experimental conditions such as influent concentration, flow rate, test duration, and column configuration. These factors varied substantially across studies included in this review, making it difficult to isolate intrinsic material properties from artefacts of experimental design.

**Table 9:** Log₁₀-transformed total removal capacity (*Sₘ*), expressed as cumulative mass removed per unit filter material (mg kg⁻^1^) during experimental studies (mean ±1 standard deviation). *Sₘ* reflects the combined effects of adsorption, filtration, biological degradation, and other removal processes. Washed excavated material and glacial till were excluded due to insufficient data

Technical criteria	Crushed rock	Crushed concrete	Shredded tires	Bark	Biochar	Natural gravel
Organic material	2 (±2)	2.2 (±0.7)	4 (±3)	4.0 (±0.1)	4 (±2)	4 (±1)
Bacteria and viruses	4 (±1)	3.2 (NA)	5 (±1)	3 (±2)	4 (±1)	4 (±3)
Nitrogen	3.15 (±0.04)	1 (±1)	5 (±4)	2.5 (±0.1)	1 (±3)	2 (±2)
Phosphorus	2.20 (±0.04)	0.8 (±0.8)	4 (±4)	1.4 (±0.7)	2 (±1)	2 (±1)
Metals	NA	NA	NA	NA	−1.1 (±0.3)	NA
PM	−2 (±1)	NA	NA	−0.6 (±0.3)	−1.1 (±0.6)	−2 (±1)
P	NA	NA	NA	1 (±2)	−0.7 (±0.4)	−0.7 (±0.3)
PB and PBM	NA	NA	NA	−1 (±1)	−0.3 (±0.6)	0.1 (±0.1)

*P*, persistent; *B*, bioaccumulative; *M*, mobile

Values represent log₁₀(mg kg⁻^1^). Negative values indicate cumulative removal below 1 mg kg⁻^1^ during the experimental period

Despite these limitations, the calculated *S*_*m*_ values were broadly similar to those of natural gravel for most materials and pollutant groups. Shredded tires showed a potentially higher removal capacity, although this observation is based on sparse datasets and relatively short experimental durations. Bark and biochar exhibited considerable variability, reflecting both material heterogeneity and inconsistencies in the available evidence. No estimates could be derived for washed excavated material or glacial till due to insufficient data.

While *S*_*m*_ provides useful insight into potential long-term retention, its application as a comparative performance metric is limited by the lack of standardized experimental conditions across studies. Attempts to identify subsets of studies with comparable configurations were constrained by data availability and would have substantially reduced the dataset, increasing the risk of selection bias. In contrast, removal efficiency is more consistently reported and provides a more robust basis for comparison across materials under varying conditions. Removal efficiency was therefore used as the primary performance indicator in the MCA, as it allows for more consistent comparison across materials despite variability in experimental conditions.

For these reasons, *S*_*m*_ was used as supporting information to aid interpretation of long-term trends but was not included as a quantitative criterion in the multi-criteria analysis. The results nevertheless highlight substantial data gaps, particularly regarding long-duration filtration performance and breakthrough behaviour. Future research should prioritize standardized, long-term loading experiments to enable more reliable assessment of cumulative removal capacity and improve comparability between alternative filter materials.

### Environmental assessment

#### Climate impact of material production

The climate impact of producing the candidate filter materials was assessed in terms of greenhouse gas emissions, focusing on extraction and processing stages. Crushed rock was associated with the highest emissions, with an estimated average of 3.03 kg CO₂e t^−1^ (Skanska Industrial Solutions AB, 2023; Vilniaus Karjerai, 2021), resulting in a rating of moderately higher climate impact (−2) compared to natural gravel with an estimated average 0.57 kg CO₂e t^−1^ (Gunnar Holth Grusforretning AS, 2022; NCC Industry Nordic AB, 2022; NOMAS Norsk Massehåndtering Ressurs AS, 2023a; 2023b; Vilniaus Karjerai, 2021). This estimate carried moderate uncertainty, reflecting variation between countries and production methods.

Several materials were assessed as having a slightly higher climate impact (−1) than natural gravel, but the underlying reasons differed between material groups. An overview of all climate-impact scores and associated uncertainty levels is provided in Table 10. The first group includes washed excavated material, crushed concrete, and glacial till. These materials were assigned −1 largely because they typically require additional processing steps such as sorting, mixing, crushing, or washing, which increases energy use and associated greenhouse gas emissions compared with natural gravel. Reported emission values for crushed concrete and glacial till ranged from roughly 1.1 to 2.8 kg CO₂e t^−1^ (Green Vision Recycling, 2022; Kadawo 2018; Miliute-Plepiene & Sundqvist 2023; Swerock AS, 2024), although most data sources were limited and system boundaries varied. For washed excavated materials, the assessment was based primarily on expert judgement because quantitative LCA data was sparse.

**Table 10:**
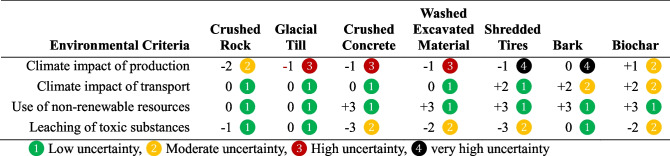
Environmental sustainability scores for the evaluated filter materials relative to natural gravel (reference score = 0), including associated uncertainty. Scores reflect relative performance based on life-cycle climate impacts, resource use, and potential environmental risks, as assessed through literature data, environmental product declarations, and expert judgment

The second group includes bark and shredded tires. These materials also received a −1 score, but for different reasons. For bark, the climate assessment is highly uncertain because there is no established LCA for bark used as a filtration material and the processing steps prior to delivery are not well defined (Elver Åkesson, Pers. comm. 2023-07−04). For shredded tires, available emission data relates mainly to the production of rubber granulate for other applications (Johansson 2018). These data suggest relatively high upstream emissions, but they are not directly transferable to the simpler cutting and washing processes expected for tire chips used in soil treatment systems. As a result, the uncertainty for both materials is substantial.

Biochar was the only material evaluated to have a lower climate impact (+1) than natural gravel (Azzi et al. 2022; Fransson et al. 2020). This rating was based on biochar’s function as a carbon sink, with life cycle studies suggesting that its use can lead to net-zero or even negative emissions, assuming stable long-term storage of fixed carbon. However, due to a lack of direct comparability with gravel production processes, the uncertainty was rated moderate.

Overall, while several materials present promising circular or low-impact alternatives, comprehensive and harmonized life cycle assessments would improve the confidence in comparative climate impact evaluations.

#### Climate impact from transport

This criterion considers the climate impact from transport of the filter material after any necessary processing has been completed. The climate impact from transport was estimated based on the bulk density of each material, under the assumption that lower density results in lower fuel consumption per transported volume. The current number of production sites and their geographic location have not been considered in the assessment, as these factors may change depending on future demand.

Bark, biochar, and shredded tires were all assessed to have a moderately lower climate impact from transport compared to natural gravel (score +2) due to their significantly lower bulk densities. However, uncertainty levels were moderate for bark and biochar due to variability in reported densities, whereas shredded tires were associated with low uncertainty thanks to well-defined physical properties.

The remaining materials, including crushed rock, crushed concrete, washed excavated material, and glacial till, were considered to have climate impacts from transport equivalent to natural gravel (score 0). This was based on similar bulk densities and well-characterized material properties, resulting in low uncertainty across all four assessments. While some processing of glacial till might slightly alter its density, this effect was considered negligible for the purposes of this evaluation.

#### Use of non-renewable resources

The use of non-renewable resources was evaluated based on whether a material is derived from virgin mineral resources or from recycled or renewable sources. Crushed rock and coarse glacial till were assessed as comparable to natural gravel, as all three are non-renewable, virgin mineral resources. In contrast, materials such as washed excavated material, bark, biochar, shredded tires, and crushed concrete were considered significantly more favorable (+3). These were classified as recycled or, in the case of bark and biochar, as renewable biomass-based materials. As this assessment was based on inherent material properties and definitions, the uncertainty was considered low for all materials.

#### Risk of leaching of toxic substances and microplastics

The risk of leaching of toxic substances and microplastics from the materials themselves varied substantially between the assessed materials. Bark and coarse glacial till were evaluated as having a comparable risk to natural gravel. Bark-based media can contain trace metals depending on where the trees were grown, but the risk for this is low. Bark may also release organic compounds such as terpenes or dissolved organic carbon, but they generally do not contain persistent organic pollutants (POPs) or microplastics (Blom & Skogsfjord 2006; Niklasson 2015). Glacial till is typically mineralogically stable and has a low likelihood of releasing harmful substances (Al-Hamdan & Reddy 2006; Allen-King et al. 2005).

Crushed rock was associated with a slightly elevated risk due to potential release of trace metals from freshly exposed mineral surfaces, which depends strongly on rock type and local geology (Elmefors et al. 2016). However, practical filtration tests using crushed rock from four sources for drinking water treatment reported no detectable metal leaching (Renman & Johansson 2005), indicating that actual risks may be low when suitable rock types are selected.

Washed excavated material and biochar were assessed as having moderately higher risks. Washed excavated material can contain PAHs, petroleum residues, or heavy metals depending on the historical land use (Afzelius 2020). Biochar composition varies widely with feedstock and pyrolysis conditions, and some biochars contain PAHs, polychlorinated dioxins and furans (PCDD and Fs), or elevated metal concentration when produced under suboptimal conditions (El-Naggar et al. 2019; Fransson et al. 2020; Gustafsson et al. 2020).

Crushed concrete and shredded tires were assessed as having the highest risk. Crushed concrete may leach Cr(VI), alkali compounds, organic pollutants such as PAHs and PCBs, or residual contaminants from binders and surface treatments (Helsing 2019; Personal communication, Linus Brander, 2023-04−20). While tire chips have demonstrated good technical performance in treatment systems, their long-term environmental safety remains uncertain. Reviews highlight potential toxicity to aquatic plants and risks of leaching rubber-derived additives, including zinc, organic compounds such as benzothiazoles and PAHs along with possible microplastic particles generated through wear and fragmentation (Graça et al. 2022; Sun et al. 2025; Wik & Dave 2009; Yang et al. 2018). These concerns or risks underpin the elevated uncertainty assigned to shredded tires in the environmental criteria and highlight the need for careful sourcing and pre-use quality control when selecting alternative filter media.

The need to avoid secondary pollution from waste-derived filter materials is well documented in other treatment contexts. Reviews of constructed wetland substrates highlight that alternative media must not introduce additional contamination risks, particularly through leaching of metals or organic compounds (Yang et al. 2018). This concern is directly relevant for materials such as shredded tires, crushed concrete, bark, and biochar, where long-term leaching behavior remains insufficiently understood.

### Social assessment

#### Health and work environment risks

In terms of health and work environment risks during handling and installation, washed excavated material and glacial till were assessed as having risk levels comparable to natural gravel (score 0). These materials are not associated with additional health hazards under normal conditions, although the assessment of washed excavated material carries high uncertainty due to limited data. Shredded tires were assessed as having a slightly lower health and work environment risk compared to natural gravel (score +1), primarily because rubber material generates less dust during handling than mineral-based materials. However, protective gear is recommended due to potential injuries from residual metal components.

Several materials were assessed as having slightly higher risk compared to natural gravel (score −1). These include bark, where dry material may produce dust that can irritate the skin and respiratory tract (Enarson & Chan-Yeung 1990); crushed concrete, which may contain sharp residuals like glass or metal; and crushed rock, where exposure to respirable quartz dust during processing is a known occupational hazard (NIOSH 2002). The assessment of crushed rock carried the lowest uncertainty due to well-documented risks.

Biochar was assessed with the highest health and work environment risk among evaluated materials (score −3), reflecting several hazards described in the underlying assessment. These include dust generation and associated respiratory and explosion risks, the potential for self-ignition during handling, and the possibility of containing toxic contaminants such as PAHs and PCDD and Fs when produced under suboptimal pyrolysis conditions (Gelardi et al. 2019; Brtnicky et al. 2021). Although not all biochars exhibit these risks to the same degree, the combination of dust formation, variability in production quality, and the need for specific safety measures supports a conservative risk rating. The uncertainty was judged to be relatively low because the underlying mechanisms for these hazards are well described in the broader biochar literature. See Table 11 for an overview of the specific criteria scores and associated uncertainty levels.

**Table 11:**
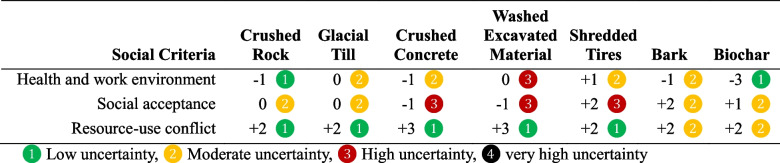
Social sustainability scores for the evaluated filter materials relative to natural gravel (reference score = 0), including associated uncertainty. Scores reflect relative performance with respect to occupational health and safety, societal acceptance, and potential conflicts over resource use, based on literature evidence, stakeholder input, and expert judgment

#### Social acceptance

Societal acceptance was evaluated from the perspective of individual property owners, focusing on the suitability of each material in soil treatment systems. Crushed rock and glacial till were considered to have acceptance levels similar to natural gravel (score 0), as both are mineral-based and already used in infiltration systems. Similarly, washed excavated material and crushed concrete were considered visually and structurally similar to natural gravel, but received slightly lower scores (−1) due to potential public concerns over contamination and lack of prior use in water treatment contexts.

Biochar and bark were assessed more positively, with scores of +1 and +2, respectively. Both are renewable, natural materials already used in private gardening, although biochar was considered less familiar to the general public. Despite their environmental benefits, a lack of measured acceptance data resulted in moderate uncertainty for both materials.

Shredded tires received the lowest score (−3) due to public concerns linked to past content of harmful substances, media coverage on microplastics from synthetic turf, and general skepticism about using rubber-based waste in household settings. The uncertainty for this assessment was high, given the lack of data specific to individual homeowners’ attitudes toward this material.

#### Resource-use conflict

The risk of resource-use conflict was evaluated based on whether the material is required for other societally important purposes. Several materials, including washed excavated material, crushed concrete, and shredded tires, were assessed as having a significantly lower conflict risk (score +3) than natural gravel. These are circular materials, not sourced through natural extraction, and are either underutilized or typically sent to landfill or energy recovery, implying that their use as filter material does not compete with more critical applications. The assessment was associated with low uncertainty due to the availability of clear data.

Materials such as bark, biochar, crushed rock, and glacial till were assigned a moderately lower risk (score +2). Bark and biochar are renewable and biobased but could become subject to increased demand in a transitioning bioeconomy, potentially creating future conflicts with other uses. Crushed rock and glacial till are mineral resources that do not impact drinking water supply like natural gravel, but their extraction still represents a physical alteration of the natural environment, especially in urban areas. These assessments carried moderate to low uncertainty, based on data availability.

No materials in the study were identified as having equal or higher conflict risk than natural gravel, reflecting a general trend toward reduced environmental and societal competition when using recycled or alternative filter media.

### Economic assessment

#### Material cost

The cost of filter materials varies considerably, with natural gravel serving as the reference point (Table 12). Several alternatives, including washed excavated materials, crushed rock, crushed concrete, coarse glacial till, and shredded tires (rubber chips), were found to have comparable overall costs. Although their raw material costs are often lower than natural gravel, additional expenses for quality assurance or modifications needed to meet filtration requirements are expected to raise their total cost to a similar level. Bark and biochar exhibited higher cost variability. Bark spans from standard decorative bark (similar in cost to natural gravel) to high-end products such as the filter substrate Zugol, which may cost up to ten times more. Biochar costs vary widely depending on production method and quality, with price estimates ranging from similar to significantly higher than natural gravel, resulting in a lower cost score. Overall, only bark and biochar were assessed as more costly than natural gravel, and both with high uncertainty due to variation in product types and lack of application-specific specifications.

**Table 12:** Indicative material costs for natural gravel and alternative filter materials, expressed as supplier gate prices in euros per tonne. Prices reflect typical Swedish market conditions and are based on publicly available supplier information, direct supplier communication, and literature sources. Costs originally reported in Swedish kronor were converted using the average exchange rate for 2023 to 2024 of 1 EUR equals 11.4 SEK. Reported ranges reflect variation in material specification, processing level, and supplier

Material	Description	Cost (EUR t^−1^)	Reference
**Natural gravel**	0–4 mm	24.50	AB Nybrogrus^a^
	0–8 mm	16.40	Heidelberg Materials^b^
**Crushed rock**	Washed	10.50–12.30	Peter Martinsson, Pers. Comm., 2024-09−19
	Washed 0–4 mm	13.60	Thomas Björklund, Pers. Comm., 2024-01−16
**Glacial till**	No specification	5.90	Rimbo Bergstäkt^c^ (quarry)
	Crushed 0–16 mm	6.80	Dalboda Naturgrustäkt^c^ (Natural gravel quarry)
	Crushed 0–16 mm	9.60	Swerock Terminal Uppsala^c^
**Excavated material**	Washed 0–4 mm	7.00–8.80	Peter Martinsson, Pers. Comm., 2024-02−28
	Washed	13.60	Mattsson 2024
**Shredded tires**	No specification	40.40	Yang et al. 2018
**Crushed concrete**	Sorted 0–4 mm	7.00–8.80	Peter Martinsson, Pers. Comm., 2024-02−28
	0–32 mm	9.00	AB Nybrogrus^a^
**Bark**	Zugol	384	Zugol, 2025^d^
	Bark 10–40 mm	33.30–34.00	Heidelberg Materials^b^
**Biochar**	For construction soils	4500–6000	Söderqvist et al. 2021
	Active biochar	395–8242	Alhashimi and Aktas 2017

^a^Data obtained from publicly available supplier information from AB Nybrogrus, accessed 15 May, 2024

^b^Data obtained from publicly available supplier information from Heidelberg Materials, accessed 15 May, 2024

^c^Direct supplier contact at Swerock Terminal Uppsala, Dalboda Naturgrustäkt och Rimbo Bergstäkt on 22 May, 2024

^d^Data obtained from publicly available supplier information from Swerock, accessed 15 May, 2024

#### Available volumes

The availability of alternative filter materials was assessed relative to current natural gravel use in small-scale soil treatment systems, estimated at approximately 84,000 m^3^ annually, with a future need of around 210,000 m^3^ per year if a 5% renewal rate is achieved. Washed excavated materials, while abundant in theory, currently have limited processing capacity (approx. 130,000 m^3^ year^−1^ from two national facilities), and were therefore rated as having moderately lower availability than natural gravel. In contrast, bark and crushed rock were considered moderately more available, with bark volumes being large due to forestry by-products and crushed rock accessible via widespread quarries, though not all sources meet filter material criteria. Crushed concrete was rated slightly more available, benefiting from nationwide construction activity, while coarse glacial till also received a moderately higher availability score due to its widespread presence. Shredded tires were rated as slightly less available, covering near-current gravel demand but constrained by prioritized recycling routes. Biochar, despite growing interest and expansion in production, remains significantly less available compared to natural gravel due to limited current production volumes and uncertain suitability of existing material for soil treatment applications. Uncertainty levels varied, with higher uncertainty typically tied to materials lacking quantification or requiring more characterization to determine usability. See Table 13 for an overview of the specific criteria scores and associated uncertainty levels.

**Table 13:**
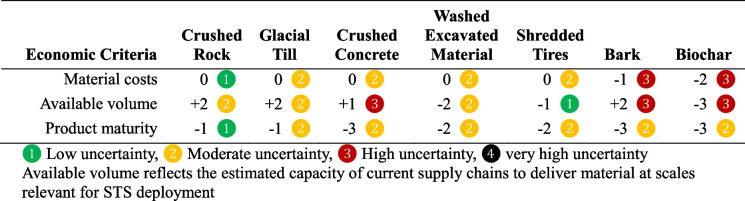
Summary of how the filter materials ranked relative to natural gravel for economic criteria, expressed as MCA scores (−3 to +3) with associated uncertainty. Scores reflect relative material cost, market availability, and product maturity under Swedish conditions

#### Product maturity

The maturity of each filter material was assessed based on its current commercial availability for water treatment applications, particularly in soil-based wastewater systems. Natural gravel serves as the reference, being a well-established and readily available product. Washed excavated materials were rated as having moderately lower maturity due to their potential suitability but current lack of quality control systems for water treatment use. Bark, biochar, and crushed concrete were all rated as having significantly lower maturity due to the absence of formal specifications, limited testing in Swedish wastewater contexts, and concerns about performance and contaminant leaching. Crushed rock and coarse glacial till received slightly lower maturity ratings, with specifications available and use in related contexts, but requiring further testing or refinement to meet application needs. Shredded tires were also considered moderately less mature, due to limited market penetration, lack of standardization, and potential health and environmental concerns, despite one known supplier. Across several materials, a common barrier to market maturity is the lack of established standards or requirements for use in small-scale soil treatment systems, which creates uncertainty and hinders broader adoption.

### Criteria weighting

While the scoring of each filter material was based on available data and aimed to be as objective as possible, the weighting of criteria represents the more subjective component of the multi-criteria analysis (MCA). The relative importance of the criteria can vary depending on stakeholder priorities and contextual considerations.

**Fig. 5:**
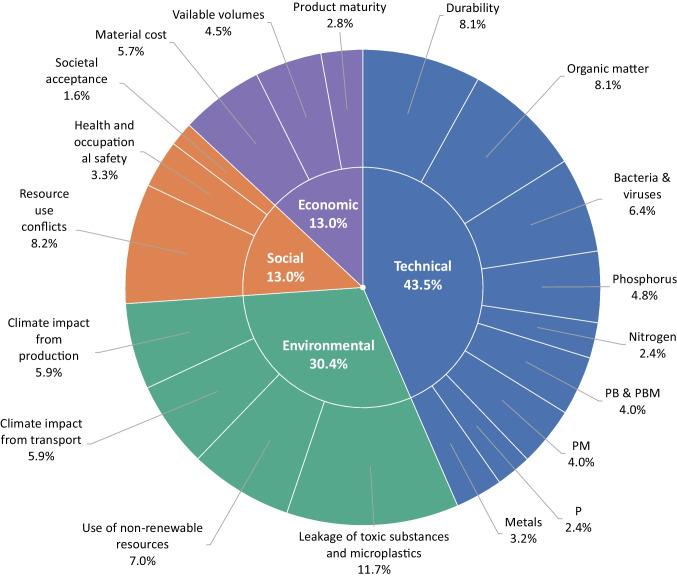
Weighting structure applied in the multi-criteria analysis, showing the relative importance of the four main criteria (inner ring) and their associated sub-criteria (outer ring). Percentages represent the final weights assigned to each sub-criterion after accounting for the weight of its parent main criterion

In this study, an initial set of weightings was developed by the project team through a structured workshop. This draft weighting was then presented to an external reference group for feedback. Based on the comments received, the weightings were revised to better reflect a broader set of stakeholder perspectives. The final weighting was designed to balance the four pillars of sustainability, technical, environmental, economic, and social, while also recognizing that certain criteria, particularly those related to technical performance, hold greater relevance in the context of replacing natural gravel in soil-based treatment systems. The final importance rating assigned to the technical, environmental, economic and social criteria were 10, 7, 3, and 3, respectively, yielding normalized weights of 43.5%, 30.4%, 13.0%, and 13.0% as illustrated in Fig. 5. 

Among the technical criteria, lifespan and the removal of organic matter were assigned the highest weights. Lifespan reflects the materials ability to maintain hydraulic and structural stability over time, which is critical for the long-term function of soil treatment systems. Removal of organic matter is a key performance indicator for aerobic treatment processes and strongly influences downstream oxygen demand. Pathogen removal received the next-highest weighting, as it represents an essential health-protection function in onsite wastewater treatment, although its relative importance is somewhat lower than the materials ability to maintain long-term operational performance.

In the social domain, resource-use conflict received the highest weighting, reflecting the growing constraint on natural gravel extraction and the need to minimize competition with drinking-water protection and other critical land uses. For the economic criteria, material cost was weighted most strongly because filter media constitute a major share of installation expenses and thus strongly influences adoption by property owners and municipalities, although availability of sufficient volumes was also recognized as important for large-scale implementation. Within the environmental category, the risk of leaching toxic substances was assigned the highest weight both within the category and across all sub-criteria, due to its direct relevance for groundwater protection, human health, and regulatory compliance. These final weightings are illustrated in Fig. 5.

The weighting approach used in this study is not intended as a prescriptive or regulatory standard but rather as a transparent and systematic framework for evaluating the potential of alternative filter materials. It enables consideration of both advantages and limitations across multiple sustainability dimensions and supports informed decision-making in future applications.

### Weighted performance scores of filter materials

**Fig. 6:**
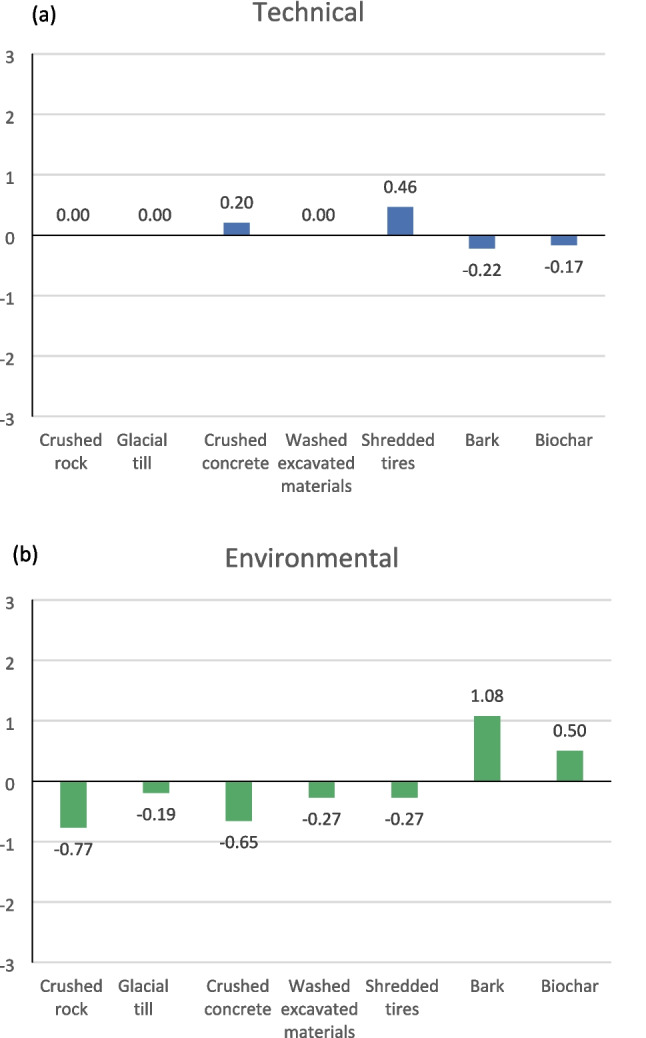

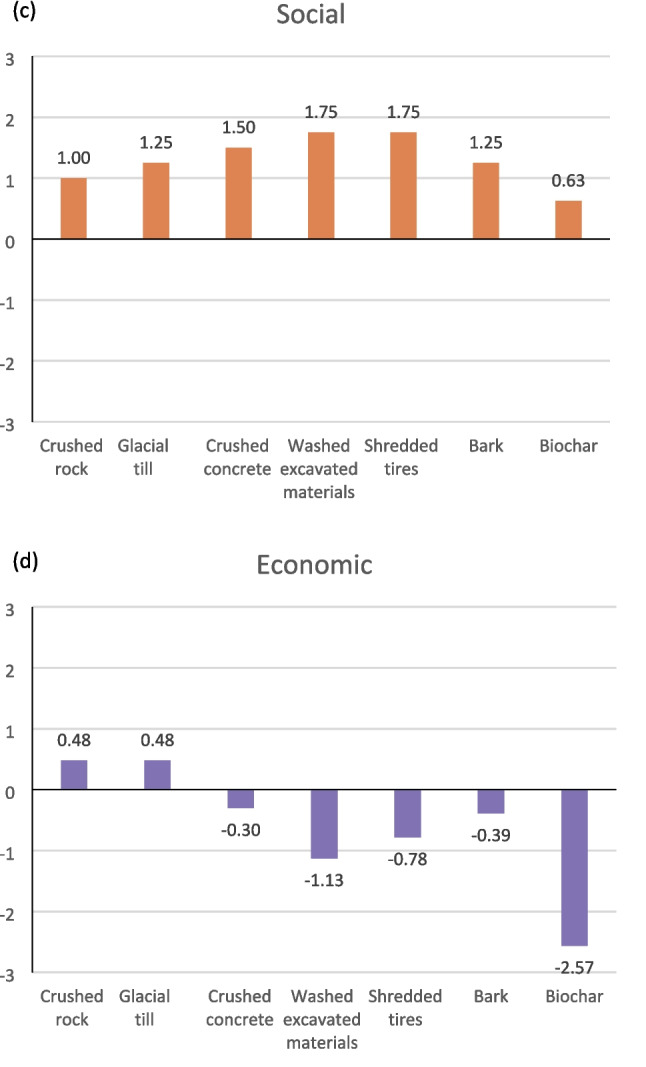
Weighted MCA scores for each filter material by main criterion: **a** technical performance, **b** environmental sustainability, **c** social acceptability, and **d** economic feasibility. Scores are expressed relative to natural gravel (0), with positive values indicating better performance and negative values indicating poorer performance. Weighting follows the scheme shown in Fig. 5

The weighted scores for the evaluated filter materials, calculated by combining the scoring results with the criteria weightings, are presented in Fig. 6. These scores provide a synthesis of the performance of each material within the four main sustainability categories: technical, social, environmental, and economic. Scores range from −3 to + 3, where positive values indicate better performance relative to natural gravel, and negative values indicate poorer performance.

The technical performance scores (Fig. 6a) show that washed excavated material, crushed rock, and glacial till achieved scores comparable to natural gravel. For washed material and till, this reflects an assumption of similar properties due to a lack of specific data. Bark and biochar scored slightly lower, primarily due to shorter estimated lifespan and reduced removal efficiency of pathogens and, in the case of biochar, metals. Metals were not among the most heavily weighted technical criteria. Shredded tires and crushed concrete scored positively, driven by good removal efficiency for organic matter, nutrients, and pathogens.

All materials scored positively within the social criteria, largely because they reduce resource use conflicts, which was the highest weighted factor in this category (Fig. 6b). Although some materials had lower scores for health risks or societal acceptance, these criteria carried lower weights, resulting in an overall positive social score.

For the environmental criteria (Fig. 6c), bark and biochar received positive weighted scores, reflecting their lower climate impact from transport and use of renewable resources. Other materials generally scored lower due to a higher estimated risk of leaching harmful substances, the most heavily weighted environmental criterion.

Within the economic criteria (Fig. 6d), all materials performed worse than natural gravel regarding product maturity. Material cost was generally comparable to natural gravel for most alternatives, except for bark and biochar, which were assessed as more expensive. Availability of sufficient material volumes contributed positively for several materials, such as bark, crushed rock, crushed concrete, and glacial till, but was a limitation for biochar, washed material, and shredded tires.

### Overall weighted scores and trade-offs

**Fig. 7:**
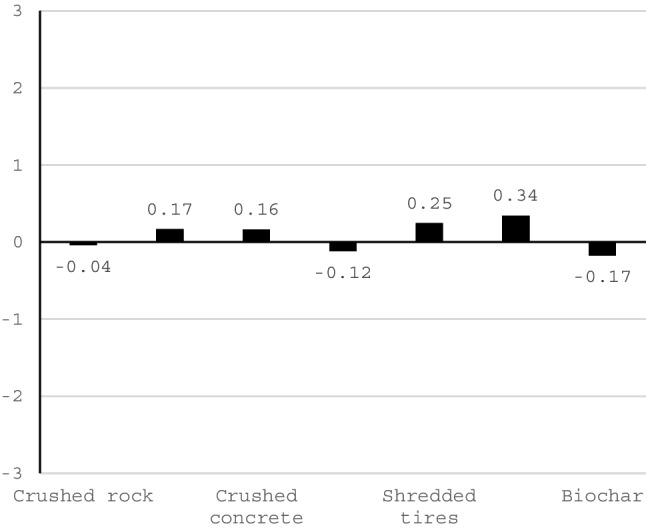
Final weighted MCA score for the seven filter materials, aggregated across all criteria (technical, environmental, economic, and social). Scores are expressed relative to natural gravel (0), with positive values indicating higher overall performance and negative values indicating lower performance

The combined weighted scores across all criteria (Fig. 7) show relatively small differences between materials, reflecting trade-offs between strengths and weaknesses in different sustainability dimensions. Bark, shredded tires, and glacial till achieved the highest overall weighted scores, while biochar scored negatively, mainly due to its low economic performance despite environmental and technical benefits.

**Fig. 8:**
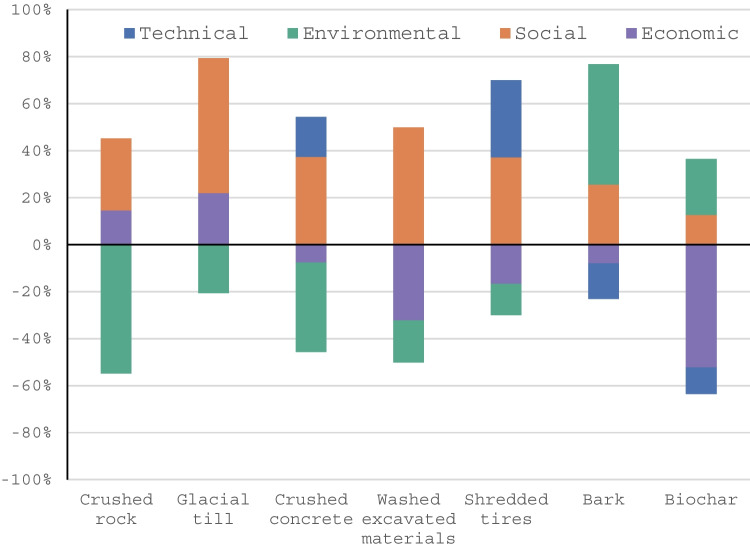
Relative contribution of the four main MCA criteria (technical, environmental, economic, and social) to the final weighted score of each filter material. Contributions are expressed as percentages of the total weighted score for each material, illustrating which criteria drive positive or negative performance relative to natural gravel (0)

A breakdown of the contribution of each main criterion to the overall score (Fig. 8) highlights these trade-offs. Bark performed particularly well in social and environmental aspects, shredded tires in social and technical criteria, and glacial till in social and economic aspects. In contrast, biochar’s negative overall score was largely driven by its economic disadvantages.

Overall, the results illustrate that all materials included in the multi-criteria analysis have potential as filter materials in soil treatment systems. Some alternatives, notably bark, shredded tires, and glacial till, emerged as more favorable options within the applied weighting framework. The findings also emphasize the importance of considering site-specific priorities and trade-offs when selecting filter materials and highlight areas where further research is needed, particularly regarding long-term performance and removal efficiency.

Overall, the weighted results show that all evaluated materials have potential to function as filter media in soil treatment systems, although their strengths and weaknesses lie in different sustainability dimensions. Bark, shredded tires, and glacial till performed most favorably under the applied weighting scheme, while materials such as biochar were penalized primarily due to economic limitations despite favorable environmental or technical attributes. These patterns underscore that the relative ranking of alternatives is shaped by inherent trade-offs between performance, environmental risk, cost, and social considerations.

Because these trade-offs vary by context and application priorities, the weighted scores should be interpreted as indicative rather than prescriptive. They highlight which materials appear most promising under current assumptions and, just as importantly, where knowledge gaps remain, particularly regarding long-term performance and treatment reliability. These considerations provide a foundation for the following section, which examines how the MCA outcomes can be interpreted in relation to practical decision-making and future research needs.

### Interpretation of MCA results

The multi-criteria analysis (MCA) provided a structured and transparent approach to evaluate the relative potential of seven alternative filter materials to replace natural gravel in soil-based onsite wastewater treatment systems. By integrating technical, environmental, social, and economic criteria, the MCA facilitated a comprehensive assessment of material performance under multiple sustainability dimensions. While the total weighted scores varied only modestly across materials, with bark, shredded tires, and glacial till achieving slightly higher scores, the analysis revealed more pronounced differences at the level of individual criteria. This underscores the importance of a multidimensional perspective when assessing sustainability-related trade-offs among alternative materials. Importantly, the MCA is not intended to dictate final material selection but rather to identify promising candidates, highlight strengths and limitations, and pinpoint knowledge gaps for further investigation.

As shown in other sustainability assessments, results from multi-criteria analysis should be interpreted as structured decision support rather than definitive rankings, since outcomes are sensitive to how criteria are weighted and valued (Neth et al. 2023). In this study, the multi-criteria analysis provided a structured and transparent approach to evaluate the relative potential of seven alternative filter materials to replace natural gravel in soil-based onsite wastewater treatment systems. By integrating technical, environmental, social, and economic criteria, the MCA enabled a comprehensive comparison of material performance across multiple sustainability dimensions.

Although some materials received notably high or low scores on individual criteria, the additive nature of the MCA methodology allowed compensatory effects, where strong performance in one domain offset weaker performance in another. For example, crushed concrete and shredded tires both received scores of −3 and +3 for certain criteria, but their net performance across all criteria was close to that of natural gravel. This indicates that while extreme scores on specific aspects can significantly influence perceptions of material suitability, these effects can be balanced when a broader sustainability perspective is applied. Consequently, most materials evaluated in this study, despite their diverse profiles, emerge as viable candidates for further consideration. It is important to highlight that minimum performance requirements, such as purification efficiency and environmental safety, were not included in the selection criteria for this study. Compliance with such requirements must be considered by relevant authorities.

The materials that achieved the highest total scores in the MCA, bark, shredded tires, and glacial till did so based on distinct strengths. Bark stood out due to its favorable environmental profile and societal acceptance, while glacial till benefited from its wide availability and low resource conflict. Shredded tires showed strong performance in technical and social domains, particularly due to their adsorption potential and low resource competition. However, these strengths are often counterbalanced by drawbacks. For instance, bark and biochar scored negatively on lifespan, while shredded tires and crushed concrete were penalized for potential pollutant leaching. These mixed profiles highlight the need for further testing and quality assurance before deployment and suggest that no single material is universally optimal, but rather, material suitability is context dependent.

### Sensitivity to weighting assumptions

To test the robustness of the weighted results, a sensitivity analysis was performed using two alternative weighting schemes:

- Equal weighting of the four main criteria categories (technical, social, environmental, economic)
- Equal weighting of all sub-criteria, regardless of category.

Despite these drastic changes, there were only minor shifts in the total scores, and the overall ranking of the materials did not change (results not shown), suggesting that the results are relatively stable under changes in weighting assumptions. However, large changes in the weighting between main criteria could potentially affect the ranking, which underscores the value of presenting performance per main category.

#### Uncertainty in criterion assessments

While the scoring and ranking of filter materials provide a structured comparison, the underlying data used for several criteria was associated with varying degrees of uncertainty, categorized into four levels (low to very high) for each criterion and material. These uncertainty estimates do not directly affect the quantitative outcomes of the multi-criteria analysis but serve as a qualitative guide to the reliability of the underlying data.

The greatest uncertainties were associated with technical criteria, particularly removal efficiency and lifespan, due to limited long-term data and a reliance on assumptions for materials like washed excavated material and glacial till. Studies of sand and gravel filters have shown that long-term performance can vary with media degradation and loading history, reinforcing the need for extended-duration studies (Hamisi et al. 2022; Martikainen et al. 2018). Other criteria with high uncertainty across multiple materials included climate impact during production, availability of material volumes, societal acceptance, and material cost.

Higher levels of uncertainty also indicate where future research should be directed, particularly to strengthen confidence in long-term performance and treatment capacity for materials with limited empirical evidence. This includes the need for standardized long-term filtration tests for bark and biochar, field-scale hydraulic and treatment evaluations of washed excavated materials and glacial till, and robust leaching studies for shredded tires and crushed concrete to clarify potential risks under realistic operating conditions.

The final evaluation also considered the interaction between high-weighted criteria and associated uncertainty. The four criteria with the greatest influence on the total scores were:

- Leaching of toxic substances and microplastics (11.7%)
- Resource-use conflict (8.2%)
- Lifespan (8.1%)
- Removal of organic matter (8.1%)

Table 14 illustrates how differences in a small number of highly weighted criteria drive the overall MCA outcomes and where uncertainty plays a decisive role. Several materials show negative scores for these key criteria, often combined with moderate to high uncertainty. Shredded tires and crushed concrete were rated negatively with respect to leaching potential, reflecting concerns related to rubber-derived additives and recycled mineral residues, respectively. Bark and biochar received negative scores for expected lifespan, indicating potential risks related to material degradation or structural stability over time. For washed excavated material and glacial till, performance with respect to organic matter removal was associated with very high uncertainty, reflecting sparse and heterogeneous data. These overlaps between high-impact criteria and elevated uncertainty identify priority areas for further research, standardization, and field validation before broader implementation can be recommended.

**Table 14:**
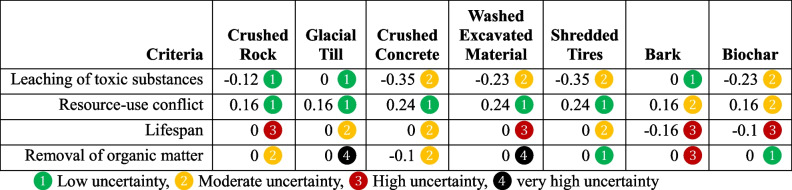
Weighted scores (*Pa,k*) for the highest-weighted criteria that contribute most strongly to differences in overall MCA outcomes, with associated uncertainty. Scores are expressed relative to natural gravel (0)

## Conclusions

This study demonstrates that reducing reliance on natural gravel in soil-based wastewater treatment systems will require a portfolio of alternative filter materials rather than a single universal substitute. All seven materials evaluated in this study, crushed rock, glacial till, washed excavated material, bark, shredded tires, and biochar, exhibited both advantages and limitations and may serve as viable alternatives under certain conditions, provided that critical technical, environmental, economic, and social performance criteria be met.

The results of the MCA suggest slightly higher overall scores for bark, shredded tires, and glacial till, although differences among materials were generally modest. Notably, bark and shredded tires were also associated with significant uncertainties, particularly with respect to long-term performance, durability, and the potential leaching of harmful substances. These uncertainties highlight the importance of cautious interpretation and context-specific evaluation when considering alternative filter media.

Across all materials, several common challenges and knowledge gaps were identified that must be addressed prior to large-scale implementation. First, some degree of material modification, such as sorting into specific particle-size fractions, is likely necessary to achieve appropriate hydraulic conductivity and treatment performance. Second, there is a clear need for comparative studies using standardized experimental setups to validate the removal efficiency of these materials under realistic conditions. Third, long-term performance data are limited, and future work should assess how filter properties may degrade over time due to material breakdown or clogging. Furthermore, robust quality assurance protocols are required to ensure consistent material properties and to minimize environmental and occupational health risks, in line with applicable regulatory frameworks. Finally, assessment of regional and national material availability is essential to ensure that alternative filter media can be supplied at a scale required to meet demand across diverse geographic contexts.

Importantly, this study does not provide a regulatory endorsement of any specific material. Instead, it presents a transparent and structured framework for evaluating alternatives filter media based on current evidence. For individual materials, further development and validation to ensure suitability, including:

- Crushed rock: assessment of the feasibility of producing suitably modified material at sufficient scale
- Glacial till: evaluation of hydraulic suitability and the need for material sorting or pre-treatment.
- Washed excavated materials: requires verification of consistent particle-size distribution and assessment of potential residual contamination from source materials.
- Crushed concrete: specification development to ensure adequate permeability and to minimize contamination risks.
- Bark: assessment of treatment performance, durability, and decomposition behavior for locally available products.
- Shredded tires: development of standardized specifications and quality control measures to ensure leaching of rubber-derived constituents remains within acceptable limits.
- Biochar: not suitable as a standalone filter medium but potentially valuable as a complementary material to enhanced removal of specific pollutants.

Overall, the applied multi-criteria analysis provides a transferable decision-support tool for identifying promising alternative filter materials in decentralized wastewater treatment systems. By explicitly accounting for performance trade-offs, uncertainty, and context-specific constraints, the framework supports informed material selection and contributes to more sustainable use of mineral resources in wastewater treatment infrastructure.

### Future perspectives

Future research should prioritize the generation of comparable, long-term performance data for alternative filter materials, particularly through standardized column and field-scale studies that capture cumulative loading, breakthrough behaviour, and durability under realistic operating conditions. Improved reporting of experimental conditions, including influent concentrations, hydraulic loading, and material characteristics, would further enhance comparability across studies and support more robust multi-criteria assessments.

From a practical perspective, implementation of alternative materials requires validation under site-specific conditions, including hydraulic performance, climate influences, and variability in material quality. Field-scale demonstration projects and long-term monitoring are therefore essential to confirm laboratory findings and to identify operational challenges such as clogging, material degradation, and potential leaching risks.

From a sustainability perspective, future work should further explore trade-offs between resource efficiency, environmental impacts, and technical performance, particularly for recycled and bio-based materials. This includes improved life cycle data, assessment of local availability and transport impacts, and evaluation of regulatory and societal acceptance barriers. Together, these efforts would support the development of more reliable and sustainable alternatives to natural gravel in soil-based wastewater treatment systems.

## Supplementary Information

Below is the link to the electronic supplementary material.

ESM 1 (XLSX 321 KB)

## Data Availability

The data used in this study are derived from published literature, publicly available reports, and expert assessments as described in the Methods section. All data supporting the findings of this study are included in the article and its supplementary material. No new primary datasets were generated during the current study.
